# Label-Free Multiphoton Microscopy for the Detection and Monitoring of Calcific Aortic Valve Disease

**DOI:** 10.3389/fcvm.2021.688513

**Published:** 2021-06-11

**Authors:** Ishita Tandon, Kyle P. Quinn, Kartik Balachandran

**Affiliations:** Department of Biomedical Engineering, University of Arkansas, Fayetteville, AR, United States

**Keywords:** calcific aortic valve disease, multiphoton microscopy, valve interstitial cell metabolism, aortic stenosis, early diagnosis

## Abstract

Calcific aortic valve disease (CAVD) is the most common valvular heart disease. CAVD results in a considerable socio-economic burden, especially considering the aging population in Europe and North America. The only treatment standard is surgical valve replacement as early diagnostic, mitigation, and drug strategies remain underdeveloped. Novel diagnostic techniques and biomarkers for early detection and monitoring of CAVD progression are thus a pressing need. Additionally, non-destructive tools are required for longitudinal *in vitro* and *in vivo* assessment of CAVD initiation and progression that can be translated into clinical practice in the future. Multiphoton microscopy (MPM) facilitates label-free and non-destructive imaging to obtain quantitative, optical biomarkers that have been shown to correlate with key events during CAVD progression. MPM can also be used to obtain spatiotemporal readouts of metabolic changes that occur in the cells. While cellular metabolism has been extensively explored for various cardiovascular disorders like atherosclerosis, hypertension, and heart failure, and has shown potential in elucidating key pathophysiological processes in heart valve diseases, it has yet to gain traction in the study of CAVD. Furthermore, MPM also provides structural, functional, and metabolic readouts that have the potential to correlate with key pathophysiological events in CAVD progression. This review outlines the applicability of MPM and its derived quantitative metrics for the detection and monitoring of early CAVD progression. The review will further focus on the MPM-detectable metabolic biomarkers that correlate with key biological events during valve pathogenesis and their potential role in assessing CAVD pathophysiology.

## Introduction

Calcific aortic valve disease (CAVD) is the most common heart valve disease, with a prevalence of 25% in those 65 years and above (1). Additionally, 75% patients with congenital bicuspid aortic valve disease develop CAVD by the age of 30 years (2, 3). CAVD is a progressive disease with complex pathophysiology (4), and is associated with a 50% elevated risk of fatal cardiovascular pathologies resulting in more than 15,000 deaths annually in North America alone (5). The only available standard of care is valve replacement surgery (6), as early detection, prevention, and mitigation strategies are underdeveloped (7). Several techniques such as echocardiography, cardiac magnetic resonance imaging, and computed tomography are clinically employed for the diagnosis and monitoring of CAVD (8, 9), while newer techniques such as positron emission tomography are gaining traction (9). However, there remains a need for a multimodal technique capable of performing early detection and monitoring of CAVD progression.

Multiphoton microscopy (MPM) is well-suited to provide non-invasive assessments of tissue structure and function. The most commonly used MPM technique, two-photon excited fluorescence (TPEF) microscopy, employs the use of two photons of near-infrared (NIR) wavelengths to excite fluorophores, which offers advantages such as intrinsic depth sectioning, less photobleaching, and label-free imaging of various endogenous fluorophores (10–13). These characteristics make TPEF suitable for non-destructive, non-invasive, spatiotemporal imaging of live cells and tissue samples, both *in vitro* and *in vivo* (10, 12, 13). MPM has been shown to be useful for generating high-resolution image-based data but also quantitative metrics that can be correlated with biologically-relevant features and events ranging from sub-cellular scales up to gross tissue morphology (10, 13–18).

TPEF allows for quantification of the endogenous fluorescence of the cellular co-factors flavin adenine dinucleotide (FAD) and the reduced forms of nicotinamide adenine dinucleotide (NADH) and nicotinamide adenine dinucleotide phosphate (NADPH). NAD(P)H and FAD play key roles as electron carriers in various metabolic pathways, including glycolysis, the tricarboxylic acid cycle, and the electron transport chain in mitochondria (11, 12, 19, 20). The ratio of the fluorescence intensity of these factors [e.g., FAD/(FAD+NAD(P)H)], called optical redox ratio (ORR), can reveal insights into the interplay between glucose catabolism and oxidative phosphorylation (11, 12, 19–21). NAD(P)H autofluorescence can also be used to assess the mitochondrial organization via the mitochondrial fractal dimension (FD) parameter (20, 22, 23).

In addition to spatially-resolved fluorescence measurements of NAD(P)H, measurements of the time between excitation and emission can provide additional insights into cell metabolism through fluorescence lifetime imaging (FLIM). This technique involves estimating the fluorescence lifetime decay rates, which are sensitive to microenvironmental changes such as pH or protein binding (11). Of note, FLIM of NAD(P)H can be used to distinguish its bound and free states through a biexponential least-squares fit of the lifetime decay curves (11, 19, 24–26). Free NAD(P)H has a mean lifetime of 0.3–0.4 ns, while protein-bound NAD(P)H has a mean lifetime of 1.9–5.7 ns (11). The proportion of free NAD(P)H tends to increase when cells are undergoing glycolysis, while bound NAD(P)H often increases with increases in the rate of oxidative phosphorylation. FLIM is advantageous because it is independent of fluorophore concentration, laser intensity fluctuations, and the effects of tissue adsorption and scattering (18, 19, 24–27).

Second harmonic generation (SHG) imaging is another powerful MPM technique used to assess non-centrosymmetric molecules like fibrillary collagen (17, 28–30). SHG is sensitive to collagen fiber amount, length, diameter, density, and orientation (31–33) and has been used in various research applications including dermatology, oncology, neurology, and cardiovascular disorders (17, 28–34). Apart from measuring collagen via SHG, TPEF imaging has been used to assess elastic fiber content, density, and length to characterize cardiovascular pathophysiology (17, 30, 35). MPM-based coherent Raman imaging techniques, such as coherent anti-Stokes Raman spectroscopy (CARS), have also provided a powerful tool to visualize lipid droplet organization, concentration, and size (24–26, 29, 36–38). SHG and CARS imaging of collagen, calcium, and lipids can be performed simultaneously with TPEF microscopy (11, 13, 20, 39, 40) and could be potentially used to characterize optical signatures associated with CAVD progression.

There are relatively few studies that have applied MPM for the study of aortic valves and its pathophysiology. MPM-based approaches combining TPEF, SHG and CARS, for label-free imaging of an aortic valve have been previously demonstrated (41). TPEF has been used to assess aortic valve interstitial cell (VIC) proliferation (21, 42), osteogenesis (43), and valve calcification *in vitro* and *ex vivo* (39). TPEF autofluorescence ratios have shown potential in assessing CAVD progression *ex vivo* (44). SHG has also been used to quantify collagen remodeling in valve tissues (45). This review outlines how label-free MPM metrics have been employed to assess key events of CAVD progression, in valvular and non-valvular cells, tissues, and disease models. We then summarize the challenges and future directions for MPM as a tool to study valve disease.

## MPM-Based Detection of Markers for Valve Disease in Other Pathologies

Multiple events contribute to CAVD progression. Some known hallmarks or markers of CAVD include endothelial damage, endothelial-to-mesenchymal transformation, oxidative stress, lipid deposition and oxidation, inflammation, collagen remodeling, and mineralization (4–7, 46–48). Multiple studies have employed label-free MPM techniques and metrics to assess similar events and biomarkers in other diseases and models as discussed below.

### Monitoring of Inflammation and Reactive Oxygen Species

Multiple studies as described here have employed multimodal MPM approach by combining CARS, TPEF, and SHG to assess inflammation. NAD(P)H imaging via TPEF has been shown to be useful in identifying macrophages and CARS has been used to detect foam cells during spinal cord injury (38, 49, 50). MPM has also been used to assess inflammation in blood vessels to identify morphological differences between healthy and tumor tissues, lymphocytes, collagen fiber bundles, and endothelial damage (51, 52). Reactive oxygen species (ROS) are a primary cause of endothelial damage and tissue injury leading to inflammatory diseases (53), and are key modulators of cell metabolism (54). Correlation between cell metabolism and ROS has been well-characterized in cancers (55). Given ROS-mediated inflammation and lipid oxidation are key drivers of early CAVD initiation (56–58), imaging metabolic and morphological changes of cells as well as oxidized lipids via MPM to infer inflammation (24, 59), may serve as a powerful early detection tool to detect CAVD initiation.

### Monitoring of Extracellular Vesicles and Apoptotic Calcification

Immune signaling, apoptosis, and Ca^2+^ ion flux are closely associated with mitochondrial dysfunction and CAVD progression (60). Apoptotic VICs have been shown to present ectonucleotide pyrophosphatase/phosphodiesterase-1 (eNPP1)-containing extracellular vesicles (EVs) on their cell membrane, where these EVs are thought to promote mineralization (61). Recently, label-free FLIM has been utilized to image EVs isolated from macrophages and cancer cells (62). NAD(P)H lifetime determined by FLIM has also been used to assess apoptosis and shown to change before cleaved caspase-3 activation and mitochondrial dysfunction (27, 63). These results suggest a potential avenue for using FLIM to characterize apoptosis, and therefore EV-mediated dystrophic mineralization during CAVD progression.

### Monitoring of Extracellular Matrix Structure and Phenotypic Differentiation

MPM has been used to monitor the osteogenic, adipogenic and chondrogenic differentiation of human mesenchymal stem cells (hMSCs) using various MPM-based metrics, including ORR, mitochondrial organization within the cell, collagen SHG, and FLIM of NAD(P)H and FAD (19, 20, 64). These studies suggest that the assessment of the heterogeneity of the cell population, their capability for collagen synthesis and remodeling, and variation in their differential potential can be assessed via MPM techniques (20, 64, 65). Considering the utility of TPEF and CARS to detect adipogenic differentiation of hMSCs and assess lipid amount, organization, orientation, and concentration (20, 25, 26, 36, 64), MPM-based imaging of lipid deposition may be useful in monitoring CAVD progression. MPM imaging has already been used to visualize elastin and collagen microstructures in heart valves using TPEF and SHG, respectively (17, 30, 45, 66).

## MPM in Aortic Valve Pathology and Physiology

### *Ex vivo* Characterization of Calcification

TPEF autofluorescence at 800 nm excitation and 460 and 525 nm emission was associated with mineralization in ApoE^−/−^ mice, calcified human valves, and calcific nodules generated *in vitro*, using a ratiometric approach, a result also corroborated by CARS imaging (39). While the fluorescence emission at 525 nm was associated with mineralization, spectral analysis revealed that fluorescence emission at 460 nm was associated with collagen (39).

**Figure 1:**
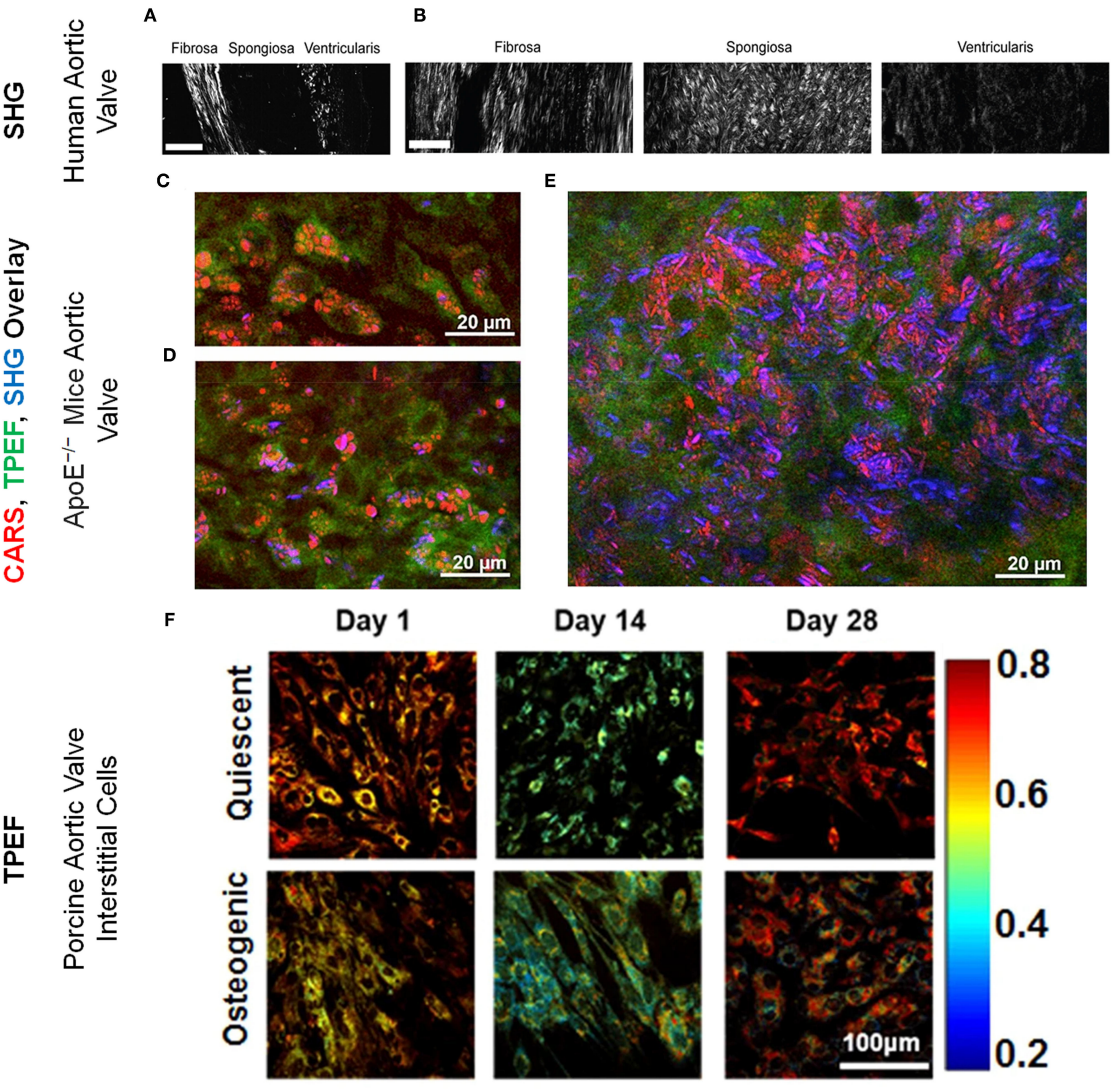
Collagen microarchitecture in **(A)** healthy and **(B)** diseased aortic valve leaflets imaged via SHG imaging. Scale bar = 50 μm. These figure panels were adapted from Hutson et al. (45) under an open access Creative Commons CC BY license. Aortic valve (acoronary) leaflet regions of ApoE^−/−^ mice imaged via CARS (red; lipids and cholesterol), TPEF (green; cells) and SHG (blue; collagen) showing **(C)** lipid droplets and **(D)** cholesterol crystals within the cells and **(E)** lipid droplets and cholesterol crystals in the plaques. These figure panels were adapted from Jannasch et al. (66) under an open access Creative Commons CC BY license. **(F)** ORR maps for VICs cultured under quiescent and osteogenic conditions for 1, 14, and 28 days. ORR decreased in osteogenic VICs by 14 days. The color bar represents the value of unitless ORR ranging between 0.2 and 0.8. These figure panels were modified from Tandon et al. (43) under an open access Creative Commons CC BY license.

Recently, we have evaluated multiple ratios of autofluorescence intensity at various stages of disease in a mouse model of CAVD (44). In that study, autofluorescence intensities at specific two-photon excitation and emission wavelengths, represented as A_excitation/emission_, were considered, including A_810/525_, A_810/460_, A_860/525_, and A_755/460._ We found that the [A_860/525_/(A_755/460_+A_860/525_)] autofluorescence ratio rather than [A_810/525_/(A_810/460_+A_810/525_)] was more sensitive to CAVD progression (44). These TPEF autofluorescence ratios correlated negatively with proliferation, osteogenic differentiation, collagen remodeling, and calcium deposition. Indeed, reduced autofluorescence ratio at 16 weeks served as a predictor for increased calcification in the valve at 28 weeks (44). In another study, assessment of collagen remodeling via SHG revealed that during CAVD, collagen fibers in the spongiosa layer increased in number, width, and density while collagen fibers of the fibrosa became relatively shorter (45) (Figures 1A,B). SHG imaging also revealed decreased collagen amount and altered fiber alignment in different regions of aortic valve leaflets in an ApoE^−/−^ mice based CAVD model (66). In the same study, lipid droplets (Figure 1C) and cholesterol crystals were identified within cells (Figure 1D) and plaques (Figure 1E) in aortic valve leaflets via CARS imaging (66).

### *In vitro* Characterization of VIC Pathophysiology

Our lab has previously reported that when VICs underwent a pathogenic phenotype shift, they experienced a decrease in ORR, suggesting a possible link between VIC pathology and its metabolic state (21, 42, 43, 67). TPEF imaging of VICs under quiescent and osteogenic conditions revealed that the ORR decreased during early osteogenic differentiation (Figure 1F) and correlated with gene expression of osteogenic markers. However, FD, a marker inversely proportional to mitochondrial clustering, increased at later time points and correlated with gene expression of osteogenic and structural markers as assessed by qRT-PCR. FD also correlated with nuclear morphology which was assessed via TPEF fluorescence maps (43). In another study, VICs subjected to conditions mimicking hypertensive pressures exhibited decreased ORR (21). Even in a more valve mimetic three-dimensional environment with physiological and pathological stretch, VIC ORR correlated negatively with proliferation (42). These studies demonstrate the *in vitro* utility of TPEF in assessing VIC structure, function, and phenotype during CAVD progression.

## Biological Relevance of MPM-Based Metrics to Valve Disease

During disease, stress, differentiation, or other pathophysiological conditions, cells undergo increased glycolysis resulting in increased production of NAD(P)H (11, 14, 20). Under physiological conditions, it has been observed that cells are preferential to the process of oxidative phosphorylation for meeting their energy demands, which results in an increase in NAD+ concentrations (11, 14, 20). This ratio of NAD+ and NADH is correlated with the ratio of FAD and NADH autofluorescence as measured by ORR (11, 20). NAD(P)H fluorescence can also be used to assess mitochondrial organization, which has been associated with multiple cardiovascular disorders (20, 23, 63, 68–73).

### Mitochondrial Organization in Calcified Valves

Dynamin-related protein-1, a protein responsible for mitochondrial fission was upregulated in stenotic valves, inhibition of which attenuated calcification (60, 74). Mitochondrial fission is known to induce autophagy (75), and inhibiting fission reduced mitochondrial clustering (72). Interestingly, osteogenic VICs showed higher FD implying less mitochondrial clustering as assessed by TPEF imaging of NAD(P)H fluorescence (43) but the specific functional role of mitochondrial clustering in valve pathophysiology is yet to be elucidated.

### CAVD Metabolic Profiling via Multi-Omics Approach

The role of cellular metabolism has been widely explored in various cardiovascular disorders (71, 73, 76, 77) and metabolic regulators like osteocalcin, pyruvate dehydrogenase kinase, and adenosine monophosphate-activated protein kinase pathway have been implicated (78–81). These factors and pathways are known to be differentially regulated in diseased aortic valves (82–84). In turn, aortic stenosis has been a known modulator of cardiac metabolism (85). Few studies exist that directly aimed at understanding metabolic changes and their role in CAVD progression (21, 42, 43, 67), while others employed proteomics and found differentially regulated metabolism-related proteins and/or protein clusters associated with aortic valve disease (86–90). Researchers have also employed transcriptomics (83, 91) and multi-omics (92) approaches and revealed differentially expressed factors, which affect cellular metabolism during aortic valve disease.

### Metabolic Changes During VIC Pathophysiology

VIC and hMSC osteogenic differentiation showcased similar trends with respect to ORR and FD (20, 43), an observation supported by the recently elucidated stemness characteristics of VICs (65). A glycolytic shift of metabolism during osteogenic differentiation has been speculated to occur due to the increased biosynthetic demand for collagen synthesis and remodeling (20, 67). However, other factors like pyruvate availability in media may also determine the tendency of cells to prefer glycolysis vs. oxidative phosphorylation (19, 20, 64). In our *in vitro* study, increased proliferation associated with decreased ORR in VICs was regulated by the Akt/mTOR signaling pathway (42), which has also been observed in other studies (55, 93). These signaling pathways were also involved in the regulation of ROS-mediated oxidative stress and its effects on cell metabolism and proliferation (54). Reduced ORR suggested increased glycolysis and/or reduced oxidative phosphorylation during VIC proliferation and osteogenesis (11, 20, 42, 43). Indeed, VICs undergoing mineralization were enriched in proteins responsible for maintaining glycolysis and mediators for phosphate metabolism (94). Additionally, peripheral blood gene signatures associated with CAVD revealed increased proliferation and reduced oxidative phosphorylation (95). Additionally, ATPase, an enzyme important for oxidative phosphorylation, was found to be downregulated in stenotic valves (83). Differences observed in the substrate utilization and glycolytic shifts in cardiovascular development and disorders (77, 85), and the extensive MPM-based assessment of hMSCs (19, 20, 64), should inspire further exploration of metabolic imaging in VICs.

Cause-effect relationships between metabolic alterations and VIC pathophysiology and their correlation with disease require further inquiry. On that front, mechanisms, regulators, and diagnostic strategies for vascular inflammation and calcification are at a more advanced stage of being assessed via imaging techniques (59, 79, 80, 96, 97), and efforts to incorporate these associations with valvular calcification may help to get a better understanding of metabolic imaging in CAVD. Further understanding of how metabolic regulators affect MPM metrics with respect to disease stage and severity, in the context of the aortic valve, is required to fully understand the extent of the biological relevance of MPM metrics in the context of CAVD.

## Challenges Associated With Clinical Translation and Future Directions

Despite several advantages, MPM is not devoid of associated limitations. Challenges exist in terms of specificity and resolution for a given fluorophore, dependency of depth penetration on the absorbing and scattering fluorophores, and minimal but existent photobleaching (11, 98). Some of the challenges associated with heart valve imaging are summarized below.

Intrinsic sources of contrast from NAD(P)H, FAD, lipids, collagen, elastin, and mineralization can facilitate MPM-based imaging of CAVD progression (11, 39). However, their overlapping spectra within the visible range can pose challenges in relating measurements to a specific source of contrast (39, 40). Unmixing the fluorescence spectra of each of the aforementioned components within the valve will be important in understanding how these optical signatures relate to disease stage and severity. Additional studies are needed for rigorous *in vitro* and *ex vivo* screening of optical signatures correlated with each CAVD hallmark, including but not restricted to endothelial damage, infiltration of inflammatory cytokines, oxidization of lipids, apoptosis, and collagen remodeling. It should be noted that while some *ex vivo* characterization has been performed (39, 44, 45), *in vivo* analysis is yet to be realized.

MPM-based intravital imaging of the ventricular wall has been performed (15, 16, 99, 100); however, *in vivo* imaging of valves faces challenges due to accessibility, tissue movement, and blood flow (28, 97, 101–104). Limitations of tissue accessibility are being addressed by enhancing flexibility and miniaturization of microendoscopy tools, which will help facilitate preclinical and clinical translation of MPM (28, 101–103, 105). Work is also focused on developing strategies to overcome motion-based artifacts introduced by heartbeat or physiological geometry changes (104). Additionally, researchers have developed algorithms to account for the NIR signal attenuation by blood (97). Indeed, there remain several challenges before MPM-based techniques can be applied to the valve leaflets *in vivo*.

Recent advances in FLIM and CARS-based imaging of lipid bilayers, oxidized lipids, extracellular vesicles, and oxidative stress (24, 37, 62) have opened new avenues for exploring label-free signatures in CAVD. Lipid infiltration, oxidation, and biosynthesis are associated with CAVD initiation and progression (106, 107), in addition to being key regulators of metabolism (11, 20). Furthermore, hypoxia-mediated collagen remodeling and cell metabolism in CAVD (11, 108–110), can also potentially be assessed by MPM imaging (17, 30, 45, 66). MPM may also prove useful in further assessing the correlation between metabolism and mineralization by imaging cellular metabolism of VICs, EVs, and mineralization of apoptotic bodies (60, 61, 111). Understanding metabolic changes and their mechanisms during heart valve pathophysiology, may therefore open new avenues for therapeutic interventions as well, as it has in other cardiovascular disorders such as heart failure, hypertrophy, and arterial inflammation (71, 73, 76, 77).

## Conclusion

MPM offers distinct advantages such as label-free detection, quantitative measurements, reduced phototoxicity, and increased depth penetration (10–13) relative to confocal microscopy. While multiple different techniques and biochemical assays have been utilized to assess CAVD progression, most techniques require the use of exogenous labels and dyes, cellular fixation, and lysis which restrict the longitudinal monitoring of live cells and tissues, unlike MPM based imaging (58, 112, 113). MPM offers a label-free non-destructive alternative that will allow conservation of the sample, time, and resources yet providing quantitative data along with spatial mapping of these biomarkers (10, 11, 13, 25, 34). MPM-based metrics have been widely employed in cancer research (18, 114–116), stem cell research (19, 20, 64, 93), wound healing studies (14, 29, 114, 117), other cardiovascular disorders (35, 97, 118, 119) (Table 1) and have been explored for clinical translation as well (29, 115, 121, 122). While MPM-based optical signatures and quantitative metrics may hold potential in streamlining *in vitro* and *ex vivo* CAVD detection and monitoring (Table 1), much work is required in elucidating distinct optical signatures that correlate with individual disease markers. Additionally, understanding the biological relevance of these biomarkers and their associated regulators is equally important in furthering the therapeutics and diagnostics of CAVD, a disease with no drug-based therapies, or early diagnostic tests (4–7). Advancements in probe design for accessibility and challenges associated with imaging moving objects with multiple confounding autofluorescent and absorbing sources must be overcome for the successful adoption of MPM techniques and metrics for clinical imaging of aortic valves (28, 97, 101–105).

**Table 1:** Summary of label-free MPM techniques and metrics associated with CAVD progression.

**CAVD stage**	**CAVD event**	**MPM techniques and metabolic metrics**	
		**Aortic valve disease**	**Non-valve disease models**
**Initiation** (Inflammation)	Endothelial damage	–	*Blood vessels*: Overlay of TPEF (Ex: 810 nm, Em: 428–695 nm) and SHG (Ex: 810 nm, Em: 395–415 nm) (51), CARS (119)
	Macrophages	–	TPEF (presumably FAD and Lipofuscin, Em: 500–550 nm) (50), *Lipid-laden Macrophages—*CARS (52)
	Lipid deposition	–	*Macrophages—*CARS (52), *Adipogenic MSCs—*TPEF (ORR; Lack of endogenous autofluorescence with autofluorescent cells) (20) *Blood vessels—*CARS (51)
	Oxidative stress	–	FLIM (Ex: <760 nm, Em: 440–470 nm) (Long lifetime species in oxidized lipids) and CARS (24, 120)
**Progression** (VIC dedifferentiation, Fibrosis, Calcification)	Apoptosis	–	*Neurons and astrocytes*—TPEF (ORR increased) (11), FLIM (Ex: <760 nm, Em: 440–470 nm; Increase or decrease of NAD(P)H lifetime dependent on cell type) (11, 27, 63)
	Extracellular vesicles	–	*EVs from macrophages and breast cancer cell lines*—FLIM (Ex: <760 nm, Em: 440–470 nm) (62)
	Hypoxia	–	TPEF (ORR decreased) and FLIM (Ex: <760 nm, Em: 440–470 nm; free/bound NAD(P)H increased) (11, 120)
	Proliferation	TPEF (ORR decreased) (21, 42–44)	TPEF (ORR decreased) and FLIM (Ex: <760 nm, Em: 440–470 nm; NAD(P)H lifetime decreased) (11, 14)
	VIC dedifferentiation	TPEF (ORR decreased early, FD increased later time points) (43, 44)	N/A
	Extracellular matrix remodeling	*Collagen—*SHG (Ex: 890 nm, Em: 425–465 nm), *Elastin—*TPEF (Ex: 760 nm, Em: 420–460 nm), (17, 30, 45, 66)	TPEF and SHG (25, 34, 52, 105, 119)
	Calcification	*Mineralization—*TPEF (Ex: 800 nm, Em: 460 and 525 nm), and CARS (39)	–

## Author Contributions

IT and KB conceptualized the manuscript. IT, KPQ, and KB co-wrote the manuscript. All authors approved the submitted version of the article.

## Conflict of Interest

The authors declare that the research was conducted in the absence of any commercial or financial relationships that could be construed as a potential conflict of interest.

## Abbreviations

CARS: Coherent anti-Stokes Raman Spectroscopy
CAVD: Calcific Aortic Valve Disease
EVs: Extracellular Vesicles
FAD: Flavin Adenine Dinucleotide
FD: Fractal Dimension
FLIM: Fluorescence lifetime imaging
hMSCs: Human mesenchymal stem cells
MPM: Multiphoton Microscopy
NAD(P)H: Nicotinamide Adenine (phosphorylated) Dinucleotide Reduced
ORR: Optical Redox Ratio
ROS: Reactive Oxygen Species
SHG: Second Harmonic Generation
TPEF: Two-Photon Excited Fluorescence
VICs: Valve Interstitial Cells.
